# Review of American Trypanosomiasis in Southern Mexico Highlights Opportunity for Surveillance Research to Advance Control Through the One Health Approach

**DOI:** 10.3389/fpubh.2022.838949

**Published:** 2022-03-15

**Authors:** Doireyner Daniel Velázquez-Ramírez, Adalberto A. Pérez de Léon, Héctor Ochoa-Díaz-López

**Affiliations:** ^1^El Colegio de la Frontera Sur, Department of Health, San Cristóbal de las Casas, Mexico; ^2^USDA-ARS San Joaquin Valley Agricultural Sciences Center, Parlier, CA, United States; ^3^Veterinary Pest Genomics Center, Kerrville, TX, United States

**Keywords:** American trypanosomiasis, surveillance, southern Mexico, One Health research, epidemiology

## Introduction

American trypanosomiasis (AT), also known as Chagas disease, is a vector-borne zoonosis of global public health importance caused by the protozoan parasite *Trypanosoma cruzi* (*T*. *cruzi*) that in endemic areas is transmitted mainly by several triatomine bug species (1, 2), although blood transfusion, organ transplantation, oral, sexual, and congenital are other routes to acquire the pathogen (3, 4). Approximately 12,000 AT-related deaths occur annually, up to seven million people are infected by *T*. *cruzi*, and around 75 million people are at risk of infection in endemic areas, mostly in regions of Latin American (LA) countries where vulnerable sectors of the population are affected (5–7). Estimates indicate that 184,000–459,000 disability-adjusted life years (DALYs) are lost worldwide due to AT (8). Climate change, international travel, and immigration of humans that are unaware of being infected are among the factors increasing the incidence of AT in endemic and non-endemic parts of the world (9–11). Research conducted since AT was described in 1909 by Carlos Chagas in Brazil documented the complex biology and ecology underlying triatomine vector-host-*T*. *cruzi* interactions that influence the risk of human infection (12–15). Cultural practices and anthropogenic environmental influences can result in disturbances of sylvatic and domestic AT cycles that promote *T*. *cruzi* transmission to humans (16–19).

Diagnosis and treatment of AT remain challenging (20). The use of a vaccine effective against *T*. *cruzi* remains to be realized (21). Early treatment is critical to manage the 28,000 new cases of AT estimated to occur every year (22). Nifurtimox^®^ and Benznidazole^®^ continue to be effective when used in the acute stage of the disease, however in chronic AT their efficacy is limited and their use is under discussion (23, 24). Mexico is one of the American countries endemic for AT where human cases and natural infection of domestic animals and wildlife reservoirs with *T*. *cruzi* were reported initially around the middle of the last century (25, 26). Of the more than 30 triatomine species documented in Mexico, around 19 species can be infected with *T*. *cruzi* and have domiciliary or intrusive habits, which is conducive to peridomestic transmission (27–29). Following recognition as a national public health problem, AT continues to burden vulnerable sectors of the population in several parts of Mexico, particularly the southern states (30).

## Current Epidemiological Situation of AT in Mexico

**Figure 1:**
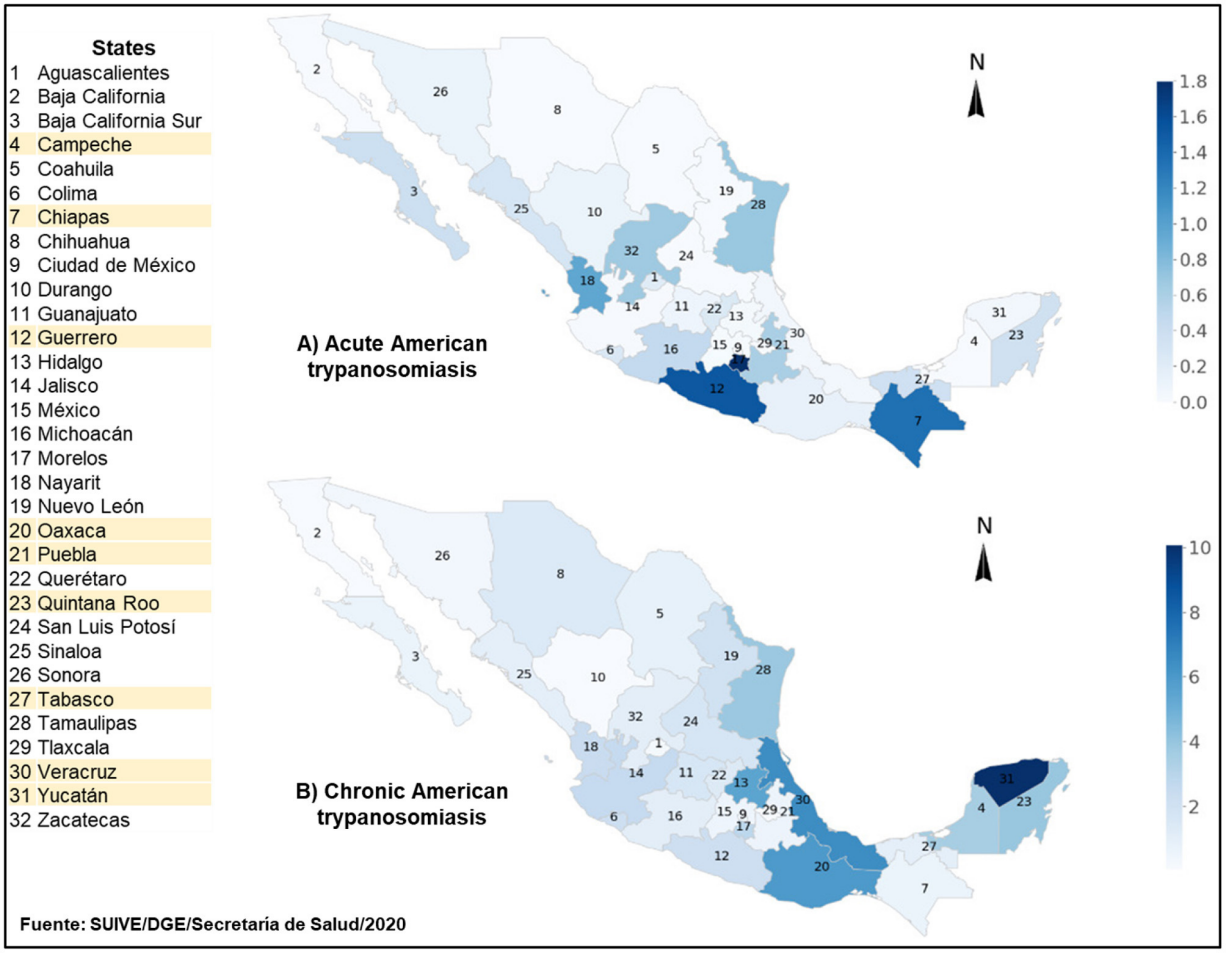
Incidence of diagnosed cases of acute **(A)** and chronic **(B)** American trypanosomiasis in Mexico for the 2017–2019 period. American trypanosomiasis (AT) incidence for each by state in Mexico is per 100 thousand inhabitants. Maps A and B depict incidence of diagnosed acute and chronic cases, respectively. The figure is based on AT data reported by the National Epidemiological Surveillance System of Mexico for the aforementioned period. South-Southeastern states are highlighted in yellow. Upper range for AT incidence in maps A and B is 1.8 and 10 per 100 thousand inhabitants, respectively.

Epidemiological data from Mexico's Ministry of Health indicate that AT is prevalent in the 32 states that make up the country (Figure 1) (31). At least two thirds of Mexico provide ecological conditions conducive for *T*. *cruzi* transmission by triatomine vectors (32). This was reflected in a 253.5% increase in the number of diagnosed cases that went from 392 to 994 between 2007 and 2016, respectively. Around 60% of the accumulated cases occurred mostly in the southern states of Veracruz, Chiapas, Quintana Roo, Oaxaca and Yucatan, and in the south central state of Morelos. Peak incidence occurred among men aged 25–49 years, largely affecting the rural population engaged in agriculture, which in some cases is an activity providing a secondary source of income (33, 34). Official data for the 2017–2019 period indicate a national upward trend in chronic AT cases (Figure 1). It is estimated that as many as four million people may be infected with *T. cruzi* in Mexico (35).

Although officially reported data increased in the last decade, the true health burden of AT in the Mexican population remains unclear because the disease remains to be fully reported (36, 37). Filling this knowledge gap is required to understand the epidemiology of AT transmission, vectorial, or non-vectorial, at the national and regional levels (38, 39). Populations in the states of Chiapas and Oaxaca are among the most affected by AT in southern Mexico. Chiapas is second nationally in acute cases of AT where an incidence of 0.43 per 100,000 inhabitants was reported in 2019 (40). The re-emergence of *Rhodnius prolixus*, one of the most efficient triatomine vectors known, complicates efforts to understand the contribution of vector transmission to the epidemiology of AT in Oaxaca, which is the state with the highest number of fatal cases recorded between 2000 and 2016 (34, 41).

## Surveillance and Control of AT in Mexico

Although AT and triatomine vectors were known to affect native civilizations in pre-Columbian times (42), the pestiferous nature of triatomine bugs to humans was chronicled by expeditionary Europeans in Mexico during the 16th century (43). *Triatoma phyllosoma* was the first triatome species now known to be a vector described from the country in the 19th century (44), and infection of a triatomine vector with *T*. *cruzi* was first reported in 1936 (45). It was until 1990 that AT was made a reportable disease in Mexico (43).

Following the proposal for official efforts based on success to control AT in other LA countries (46, 47), Mexico established the “Programa de Acción Específico para la Vigilancia, Prevención y Control de la Enfermedad de Chagas 2013–2018” (“Specific Action Program: Prevention and Control of Chagas Disease 2013–2018”) (31). Main components of this program include the disruption of vector transmission through integrated triatomine management and the elimination of *T*. *cruzi* transmission through the congenital and blood transfusion routes (32). The use of residual insecticides to control vectors in domiciles, structural improvement, and enhancement of domestic hygiene, prevention of invasion, and establishment of vectors indoors using meshed doors and windows and the implementation of bed nets to minimize the risk of human exposure to infected vectors while sleeping are planned to disrupt vector transmission (34). Continued research will yield critical information on the ecoepidemiology of AT that could be used to adapt the program to advance AT surveillance and control in Mexico.

## One Health Approach to Surveillance Research Presents an Opportunity to Advance Control of American Trypanosomiasis in the Southern States of Chiapas and Oaxaca

According to the World Health Organization (WHO), One Health is an approach where multiple sectors communicate and work together to achieve better public health outcomes (48). In this context, WHO recognizes the complexity surrounding control of neglected tropical diseases and emphasizes the need for a paradigm shift from disease-specific interventions to holistic cross-cutting approaches coordinating with other disciplines (49). The One Health approach provides the opportunity to realize this shift involving collaborative and transdisciplinary efforts to achieve optimal health outcomes among people, animals, and their shared environment to advance research for sustainable AT surveillance and control (50).

Research in context of the One Health concept was suggested as an alternative that can help understand the complexities of AT as a vector-borne disease system with diverse components under different epidemiological landscapes (51–53). Although the One Health concept resembles the Ecohealth strategy in some regards, there are differences between these approaches to address research on zoonotic vector-borne diseases (54, 55). There is room to integrate the two approaches to advance research on veterinary public health. Although Ecohealth research on AT was conducted (56), studies taking the One Health approach for research on AT remain to be designed and implemented in Mexico.

Several domestic animals and wildlife species are hosts of known triatomine vectors in Mexico (57–59). Wild mammals maintain *T*. *cruzi* in nature and facilitate its dispersion (60–62). However, information on the involvement of domestic animals and wildlife as triatomine hosts and *T. cruzi* reservoirs in the context of ecological and genetic variables is scant for the states of Chiapas and Oaxaca (63, 64). This is a significant epidemiological gap since the first clinical human cases of AT were reported in these states and they are among the most affected (Figure 1).

Applying the One Health concept to surveillance research could generate knowledge to advance AT control efforts in the southern states of Chiapas and Oaxaca through studies that investigate animal, human and environmental health as a unified theme (50). Table 1 exemplifies how consideration of the One Health domains helped identify knowledge gaps in epidemiological aspects of AT related to triatomine vector transmission in the wild, peridomestic, and domestic cycles that could be applied in the design of diverse public-private research partnerships as it was suggested to address the problem with antimicrobial resistance (65). Shared challenges for the effective implementation of One Health research initiatives yielding successful outcomes include overlapping causes and crosscutting causal relations (66, 67). Implementing One Health research on AT in Chiapas and Oaxaca considering the needs in Table 1 will require: (1) identifying common features of the pathogenic landscape between the states to investigate suspected hotspots of AT transmission (68); (2) establishing laboratory network that uses standardized molecular diagnostic tests for improved operations and rapid surveillance reporting (69); (3) developing electronic data reporting linked to information systems that update stakeholders frequently; and (4) adapting the principles of One Health epidemiological reporting of evidence (70).

**Table 1:** Application of One Health approach to identify needs for American trypanosomiasis research emphasizing surveillance in the southern states of Chiapas and Oaxaca presents opportunity to advance disease control through public-private collaboration in Mexico.

**One health domain**	**Epidemiological knowledge gap**	Research need^*^	**Facilitators of research partnerships**
Human	Qualitative (e.g., social and cultural) Quantitative (e.g., demographic, exposure factors, and behavioral)	✓ Identify risk factors ✓ Test for antibodies against *T. cruzi* in serum by serological tests (ELISA, IFA, and IHA) ✓ Diagnose *T. cruzi* genetic material in blood by molecular tools (PCR, LAMP) ✓ Xenodiagnosis of *T. cruzi* ✓*T. cruzi* detection by microscopy	**National level** • Federal human health institutions • Federal animal health institutions (wildlife, livestock) • Federal environmental institutions **State level** • State human health services • State animal health services • State environmental services **Regional and local level** • District/municipal human health services (hospitals, health center) • District/municipal animal health services • District/municipal environmental services • Research centers • Non-governmental organizations • Public and private universities • Community key actors • Community participation
Animal	Domestic mammals Free-ranging wildlife Captive exotic mammals Biological vector	✓ Diagnose *T. cruzi* genetic material in blood by molecular tools (PCR, LAMP) ✓*T. cruzi* detection by microscopy	
Environmental	Abiotic factors Biotic factors Alternate transmission	✓ Evaluate chemical and physical variables ✓ Assess biological components of the ecosystem ✓ Identify biological or inanimate fomites	

* *ELISA, Enzyme-linked immunosorbent assay; IFA, Immunofluorescence assay; IHA, Indirect hemagglutination assay; PCR, Polimerase chain reaction; LAMP, Loop-mediated isothermal amplification*.

## Conclusion

Adapting the One Health approach to research on AT is an opportunity to advance surveillance and control efforts of this neglected disease that burdens disproportionately rural and semirural populations in southern Mexico. This may be challenging in the states of Chiapas and Oaxaca where it has been argued the situation reached a crisis point and where other vector-borne diseases affecting urban populations divert attention from AT (71, 72). However, the official action plan to prevent and control AT provides the avenue for transdisciplinary collaboration involving human and animal health care professionals (31), which can facilitate the implementation of One Health research to prevent AT in rural and semirural communities. Adapting the One Health concept will augment the impact of epidemiological studies needed in Oaxaca, Chiapas and other southern states in Mexico to understand the involvement of infected domestic animal and wildlife hosts bitten by triatomine vectors involved in the sylvatic, peridomestic, and domestic cycles. Translating this research could improve molecular tests to characterize and detect *T*. *cruzi* across triatomine vector and mammalian host species and develop molecular assays for susceptibility of triatomines to insecticides and *T*. *cruzi* to drugs. Community participation could be promoted by sharing with the public a comprehensive perspective of the human-animal-environmental interface based on all this information. Realizing the cultural change required to practice One Health by public health professionals and veterinary clinicians will enable timely diagnosis and treatment of AT.

## Author Contributions

All authors listed have made a substantial, direct, and intellectual contribution to the work and approved it for publication.

## Funding

This work was funded by National Council of Science and Technology (CONACYT) Project A1-S-70901 to DV-R.

## Conflict of Interest

The authors declare that the research was conducted in the absence of any commercial or financial relationships that could be construed as a potential conflict of interest.

## Publisher's Note

All claims expressed in this article are solely those of the authors and do not necessarily represent those of their affiliated organizations, or those of the publisher, the editors and the reviewers. Any product that may be evaluated in this article, or claim that may be made by its manufacturer, is not guaranteed or endorsed by the publisher.
